# Dysregulation of testis mRNA expression levels in hatchery-produced *vs* wild greater amberjack *Seriola dumerili*

**DOI:** 10.1038/s41598-023-40597-5

**Published:** 2023-08-22

**Authors:** Anna Lavecchia, Caterina Manzari, Chrysovalentinos Pousis, Luigi Mansi, Sharon N. Cox, Constantinos C. Mylonas, Rosa Zupa, Claudio Lo Giudice, Caterina De Virgilio, Ernesto Picardi, Gianluca Ventriglia, Graziano Pesole, Aldo Corriero

**Affiliations:** 1 Department of Biosciences, Biotechnologies and Environment, University of Bari Aldo Moro, Via Orabona 4, 70124 Bari, Italy; 2 Department of Veterinary Medicine, University of Bari Aldo Moro, S.P. per Casamassima km.3, 70010 Valenzano, Bari Italy; 3 Institute of Marine Biology, Biotechnology and Aquaculture, Hellenic Centre for Marine Research, 71003 Heraklion, Crete Greece; 4 Institute of Biomembranes, Bioenergetics and Molecular Biotechnologies, National Research Council, Via Giovanni Amendola, 122/O, 70126 Bari, Italy

**Keywords:** Molecular biology, Bioinformatics, Gene expression analysis, Microscopy

## Abstract

Reproductive dysfunctions have been recently documented in male greater amberjack *Seriola dumerili* caught from the wild and reared in captivity. In the present study, we compared testis transcriptome in wild fish (WILD), hatchery-produced fish with apparently normal spermatogenesis (Normal Farmed; NormalF) and hatchery-produced fish with evident reproductive dysfunction (Dysfunctional Farmed; DysF). Gene expression analysis identified 2157, 1985 and 74 differentially expressed genes (DEGs) in DysF *vs* WILD, NormalF *vs* DysF and NormalF *vs* WILD comparisons, respectively. In DysF, a dysregulation of several interconnected biological processes, including *cell assembly*, *steroidogenesis* and *apoptosis* was found. Gene enrichment of *progesterone-mediated oocyte maturation*, *oocyte meiosis* and *cell cycle* pathways were identified in the DysF *vs* NormalF comparison. Most of the DEGs involved in the enriched pathways were downregulated in DysF. The comparison of NormalF *vs* WILD showed that most of the DEGs were downregulated in NormalF, including a gene that encodes for a regulatory protein with a protective role in apoptosis regulation (*ptpn*6), indicating that spermatogenesis was dysfunctional also in the apparently “normal” hatchery-produced fish. Hence, rearing of male greater amberjack in captivity, from eggs produced by captive breeders, did not prevent the appearance of reproductive dysfunctions, and these dysfunctions involved several biological processes and metabolic pathways.

## Introduction

Fish reared in captivity are often affected by reproductive dysfunctions of variable severity^1–3^. In general, females show oogenesis alterations which may involve incapacity of oocytes to start secondary oocyte growth, block of vitellogenin uptake before completion vitellogenesis, or failure of oocytes to undergo maturation when vitellogenesis is accomplished. In males, spermatogenesis impairment results in reduction of sperm quantity and/or quality. These gametogenesis impairments are considered to be a consequence of confinement-induced stress^4–7^, lack of the natural spawning conditions^8–10^, and/or inadequate diet^11,12^.

The greater amberjack *Seriola dumerili* is a cosmopolitan species found throughout the temperate zone, including the Indo-West Pacific Ocean^13^, the Western Atlantic Ocean^14,15^, the Eastern Atlantic Ocean and the Mediterranean Sea^16^. The available total worldwide catches data of this species are outdated and indicate a global fishery production of ≈ 3300 tonnes in 2009^17^. Due to the worldwide consumer’s appreciation and the high market quotations of this species, greater amberjack domestication represents an excellent opportunity for product diversification in aquaculture.

A few recent studies documented the occurrence of severe reproductive dysfunctions in greater amberjack of both sexes caught in the Mediterranean as juveniles and reared in captivity in marine cages for a few years until sexual maturity (see review by^18^). Compared with wild breeders, captive-reared greater amberjack males showed lower relative testicular mass (gonadosomatic index) and sex steroid plasma levels throughout the gonadal recrudescence, active gametogenesis and spawning phases of the reproductive cycle^12^. Moreover, captive-reared greater amberjack males exhibited smaller seminiferous lobules, early cessation of the active spermatogenesis phase, and high rate of germ cell apoptosis associated with abnormally high 17β-estradiol plasma concentrations during the gametogenesis recrudescence in spring. The observed reproductive anomalies finally resulted in the production of sperm of low quality, characterized by low percentage of motile spermatozoa, limited motility duration and velocity, and low ATP content^19^. Nevertheless, the documented spermatogenesis dysfunctions did not prevent the breeding of males and the production of fertilised eggs after the application of spawning induction therapies^20–22^, although the reported fertilization rate (30–45% in^20^ and 35–80% in^21^) and larval survival (5d larval survival, 5–30% in^20^ and 10–30% in^21^) were rather low and variable.

The present understanding of the endocrine mechanisms responsible for reproductive dysfunctions occurring in fish reared in captivity is limited. In many cases, the endocrine causes of gametogenesis impairment involve a reduced release (but not synthesis) of luteinizing hormone (Lh) from the pituitary^2,23–25^. In fact, the stimulation of Lh release from the pituitary through the administration of a gonadotropin releasing hormone agonist (GnRHa) has proven widely to be a useful tool to alleviate reproductive dysfunctions related to reduced sperm production and failure of oocyte maturation^26–30^, including greater amberjack^20–22,31^.

In the present study, we have undertaken a comparative analysis of testis transcriptome of hatchery-produced greater amberjack versus wild breeders sampled during the reproductive season, as part of a wider research aiming at describing the effects of captive rearing on reproductive function.

## Methods

### Ethics

For the present study, wild and farmed greater amberjack males were used. Wild fish were commercially caught from an authorized purse-seine fishing vessel during routine fishing operations. Immediately after death, male fish whose size was beyond that of first maturity^32^ were purchased and sampled on board. Farmed fish were produced from eggs obtained in Argosaronikos Fish Farm S.A. (Salamina Island, Greece) in 2017 and reared under routine farming condition. The use of the farmed fish used in the present study was approved by the Greek National Veterinary Services (AP 31337). All procedures involving animals were conducted in accordance to the “Guidelines for the treatment of animals in behavioral research and teaching”^33^, the Ethical justification for the use and treatment of fishes in research: an update^34^ and the “Directive 2010/63/EU of the European parliament and the council of 22 September 2010 on the protection of animals used for scientific purposes”^35^. The authors complied with the ARRIVE guidelines.

### Sampling

Four wild and six farmed greater amberjack males were sampled on 31 May–01 June 2021 during the active gametogenesis period of the species in the Mediterranean Sea^12^. Wild fish were caught around the Pelagie Islands (Sicily, Italy) from a purse-seine fishing vessel and sampled on board immediately after death. Farmed fish used in the present study were produced from eggs obtained in Argosaronikos Fish Farm S.A. (Salamina Island, Greece) in 2017, after spawning induction of wild-caught breeders^21,22^. The hatchery-produced (first generation, F1) juveniles were stocked at the same farm and they were maintained following common aquaculture practices. A commercial broodstock diet (Skretting, Vitalis Prima) was administered 3 to 5 times a week until apparent satiation.

Before sampling, captive-reared fish were confined in a small cage area using a PVC curtain and then were tranquilized with about 0.01 ml l^−1^ clove oil (Roumpoulakis E.P.E., Greece) dissolved in ethanol at a 1:10 ratio. Then, they were gently directed into a PVC stretcher, brought on board of a service vessel, and anesthetized deeply with 0.03 ml l^−1^ clove oil. Then the fish were euthanized by decapitation, were placed in crushed ice and transferred to the farm facility for further collection of biometric data and tissue samples. The time interval between fish death and sampling ranged between 30 min and 2 h.

For each fish, biometric data (fork length, FL, nearest cm; body mass, BM, nearest hg; gonad mass, GM, nearest g) were recorded, and the gonado-somatic index was calculated as GSI = 100 GM BM^−1^ (Table 1). Testes were excised and preserved as below specified.

**Table 1:** Greater amberjack sampling date, origin, biometric data, gonadosomatic index and reproductive state.

Sampling date	Fish origin	Fork length (cm)	Body mass (kg)	Gonad mass (g)	GSI	Reproductive state*	Group**
31/05/2021	Wild	95	9.3	50	0.5	Advanced spermatogenesis	WILD
31/05/2021	Wild	101	13.0	300	2.3	Advanced spermatogenesis	WILD
31/05/2021	Wild	92	9.2	100	1.1	Advanced spermatogenesis	WILD
31/05/2021	Wild	93	8.8	60	0.7	Advanced spermatogenesis	WILD***
01/06/2021	Farmed	81	7.9	54	0.7	Advanced spermatogenesis	NormalF
01/06/2021	Farmed	75	6.1	86	1.4	Advanced spermatogenesis	NormalF
01/06/2021	Farmed	73	8.2	71	0.9	Advanced spermatogenesis	NormalF
01/06/2021	Farmed	80	7.7	69	0.9	Advanced spermatogenesis	NormalF
01/06/2021	Farmed	76	6.8	27	0.4	Arrested spermatogenesis (spent)	DysF
01/06/2021	Farmed	91	12.4	49	0.4	Arrested spermatogenesis (spent)	DysF

GSI, gonadosomatic index. *The reproductive state was assessed as described in the “Methods” section. **For comparative transcriptome analysis, fish were grouped according to their origin and reproductive state as described in the “Results” section. DysF, reproductively dysfunctional farmed fish; NornalF, non-dysfunctional farmed fish; WILD, wild fish with normal spermatogenic activity. ***This sample did not pass the RNA quality check and was excluded from RNA-seq analysis.

### Histological analysis of greater amberjack testes and seminiferous tubule diameter

For the histological analysis of greater amberjack testes, 1-cm thick gonad slices were cut and fixed in Bouin’s solution, dehydrated in ethanol, clarified in xylene and embedded in paraffin wax. Five-μm thick sections were then stained with haematoxylin–eosin. For the assessment of the reproductive state, the type of spermatogenic cysts was recorded and the amount of spermatozoa in the lumen of seminiferous lobules was subjectively evaluated^12^.

At least 50 seminiferous tubules were selected randomly from one histological section and their diameter was measured from microphotographs taken with a digital camera (DFC 420; Leica, Cambridge, UK) connected to a light microscope (DIAPLAN; Leitz, Wetzlar, Germany). Measurements were performed using an image analysis software (Leica Application Suite, version 3.3.0, Cambridge, U.K.).

**Figure 1:**
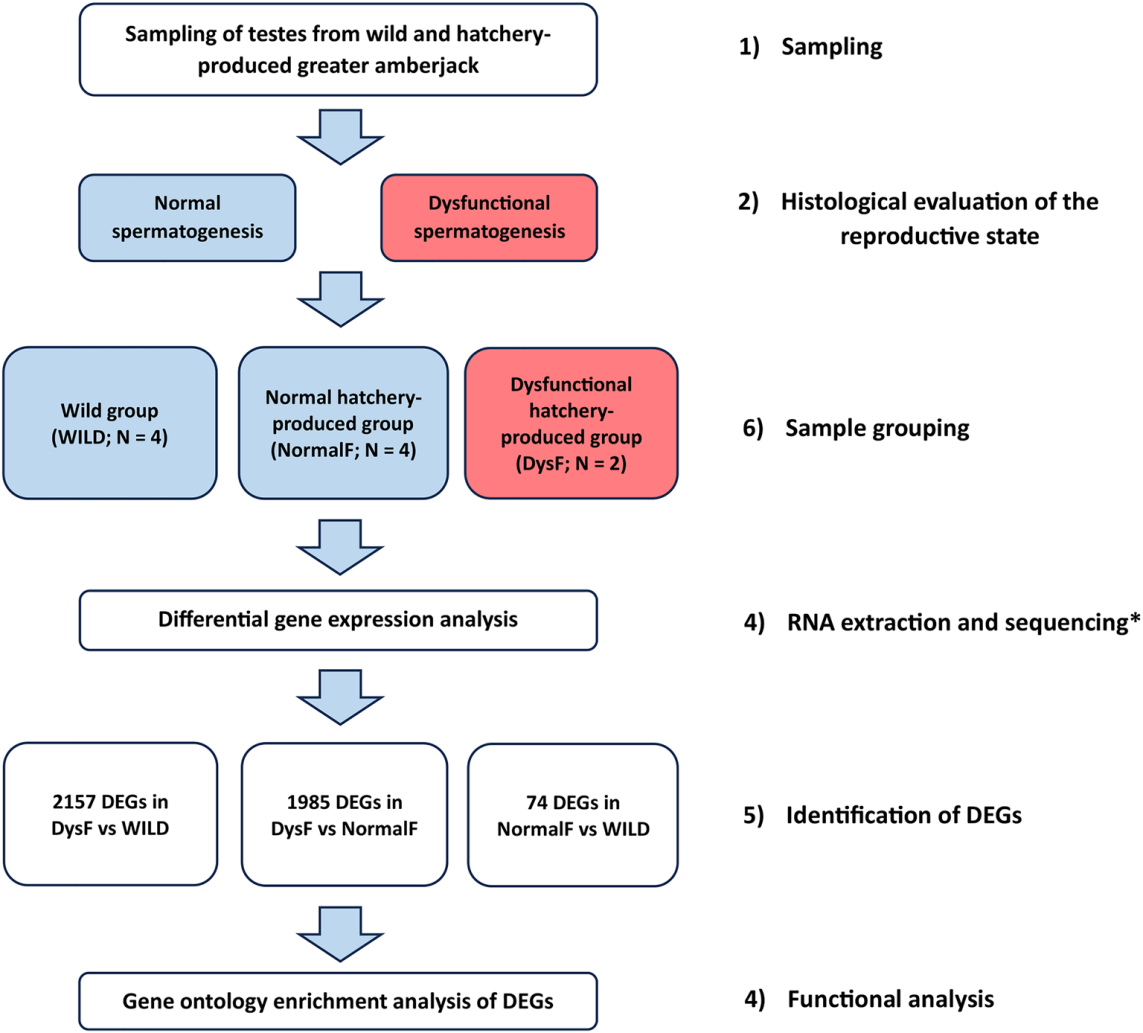
Schematic representation of the experimental design. (1) Testis samples were taken from wild and hatchery-produced greater amberjack. (2) The reproductive state was assessed through the histological analysis of the testes. (3) Fish were then divided in three groups based on their origin and reproductive state: WILD (wild fish showing normal spermatogenesis), NormalF (hatchery-produced fish showing normal spermatogenesis); DysF (hatchery-produced fish showing altered spermatogenesis). (4) Testis RNA was extracted and sequenced. (5) Differentially expressed genes (DEGs) between groups were identified. (6) Functional analysis of DEGs was carried out. * After the evaluation of the RNA quality, one of the wild samples was excluded from further analyses.

Based on the histological evaluation of the reproductive state, the fish were divided in three groups (see “Results” section) that underwent a comparative analysis of testis transcriptome (Fig. 1).

### RNA extraction and sequencing

For RNA-seq, small testis samples were stored in RNA later® (Thermo Fisher Scientific, Waltham, Massachusetts, U.S.), transported in the laboratory within one week and frozen at -80 °C. Total RNA extraction was performed on 2.5 mg testis samples, lysed and homogenised with TissueLyser II (Qiagen, Germany) setting 2 min and 20 Hz frequency, by RNeasy® Plus Micro kit (Qiagen, Germany) following the manufacturer's protocol. The quantity and quality of extracted Total RNA were checked for quantity and quality respectively by Nanodrop 1000 spectrophotometer (Thermo Scientific, Waltham, Massachusetts, U.S.) and Agilent 2100 Bioanalyzer (Agilent Technologies, Santa Clara, California, U.S.), respectively. After the evaluation of the RNA quality, one of the wild samples (Table 1, row 4) was excluded from library preparation and sequencing. All the other samples, showing high quality RNA (RIN range 7–8), were used to prepare the mRNA libraries by SureSelect Strand Specific RNA Library Preparation kit (Agilent Technologies, Santa Clara, California, U.S.). In particular, poly-A selection and directional mRNA libraries were carried out using 1 µg of total RNA. Finally, paired-end sequencing (2 × 75 bases) was performed on the Illumina NextSeq platform (Illumina Inc., San Diego, California, U.S.).

### RNA-seq data analysis

Sequencing raw data in FASTQ format, were quality-checked using the FastQC program (http://www.bioinformatics.babraham.ac.uk/projects/fastqc) and adaptor sequences as well as low quality regions (phred cutoff of 25) were trimmed using fastp (version 0.20.0) (with parameters: --detect_adapter_for_pe -x -q 25 -n 1 -l 50 -y -w 8)^36^. Cleaned reads were aligned onto the *Seriola dumerili* reference genome (Sdu_1.0, assembly accession GCF_002260705, https://www.ncbi.nlm.nih.gov/assembly/GCF_002260705.1) using STAR (version 020201)^37^ with default parameters. Read counts per gene were performed by featureCounts (version 1.6.0)^38^ and differential gene expression analysis was carried out using DESeq2^39^. Only genes with an adjusted P value ≤ 0.05, |log2(FC)|> 1.5 and |log2(FC)|< 1.5 were used for downstream analyses.

DAVID (Database for Annotation, Visualization, and Integrated Discovery database https://david.ncifcrf.gov/tools.jsp)^40^ and ShinyGO (http://bioinformatics.sdstate.edu/go)^41^ were used to perform the functional annotation of Differently Expressed Genes (DEGs) and the GO enrichment analysis. By using a False Discovery Rate (FDR < 0.05), these analyses were able to highlight specific categories (biological processes, molecular functions, cellular components and pathways), potentially involved in reproductive dysfunctions. A network based on protein–protein interaction (PPI) between DEGs associated to each comparison was built by STRING (https://string-db.org/). KEGG Mapper—Search (https://www.genome.jp/kegg/mapper/search.html) was used to explore DEGs specifically associated to apoptosis pathway^42^.

All queries launched on DAVID, ShinyGO and STRING were restricted to taxon ID 41447 (*Seriola dumerili*).

### Statistical analysis

Differences in GSI and diameter of seminiferous tubules were evaluated by a two tailed Student’s t-test between the following groups that were identified based on testis histological analysis (see “Results” section): WILD *vs* NormalF; WILD *vs* DysF; NormalF *vs* DysF. The results are presented as means ± SD; the statistical probability significance was established at the P < 0.05 level.

## Results

### Evaluation of reproductive state and samples selection for RNA-seq

**Figure 2:**
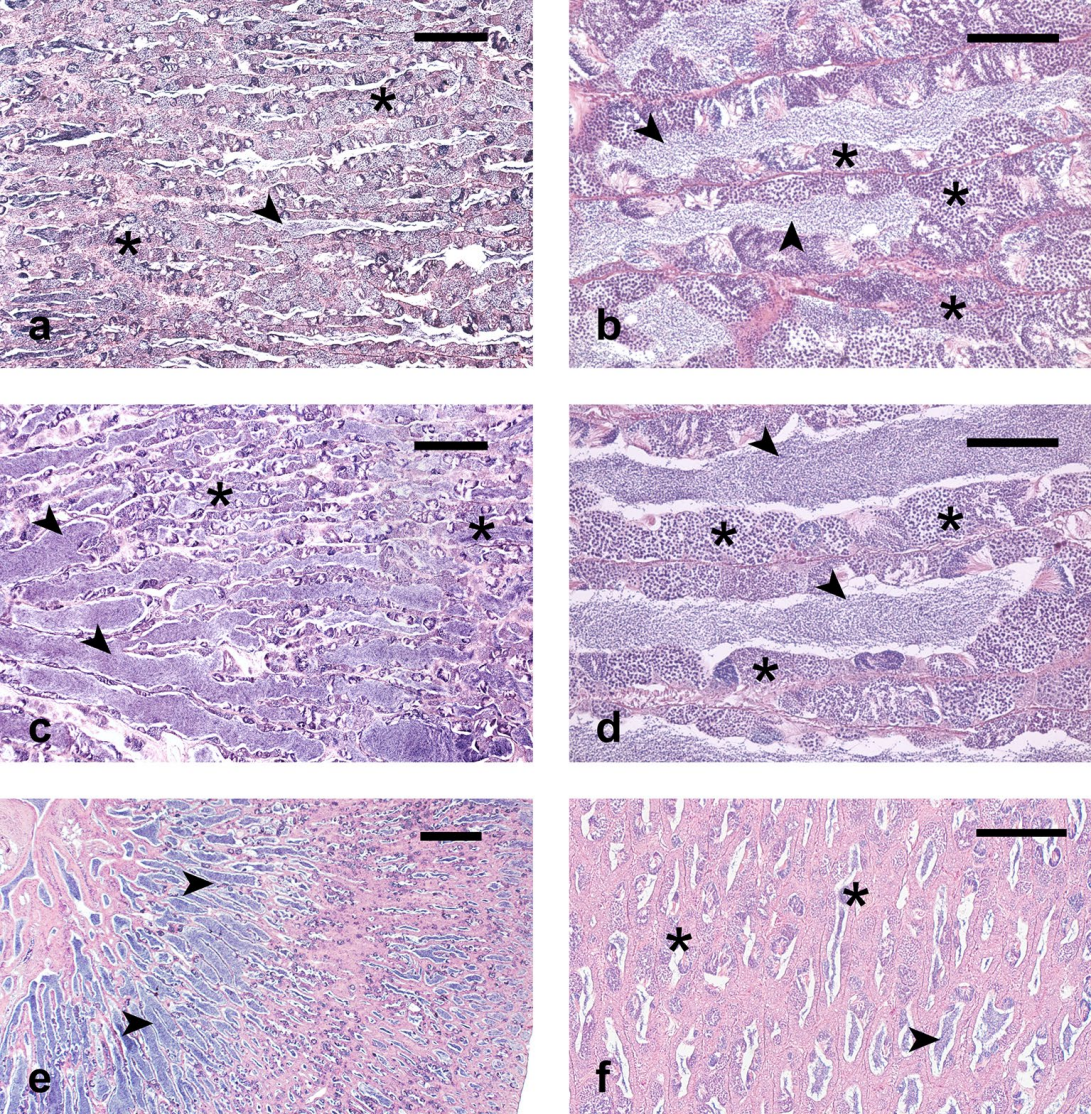
Micrographs of histological section from greater amberjack testes. (**a**, **b**) Wild specimen in active spermatogenesis phase (WILD group) showing large seminiferous tubules rich in spermatocysts. (**c**, **d**) Farmed specimen in active spermatogenesis with histological appearance similar to wild fish (NormalF group). (**e**, **f**) Farmed specimens showing arrested spermatogenesis (DysF group). Small seminiferous tubules with residual spermatocysts and luminal spermatozoa can be observed. Arrowheads indicate luminal spermatozoa; asterisks indicate spermatocysts. Hematoxylin–eosin staining. Bars = 100 μm in (**b**) and (**d**), 200 μm in (**f**), 300 μm in (**a**) and (**c**), 500 μm in (**e**).

**Figure 3:**
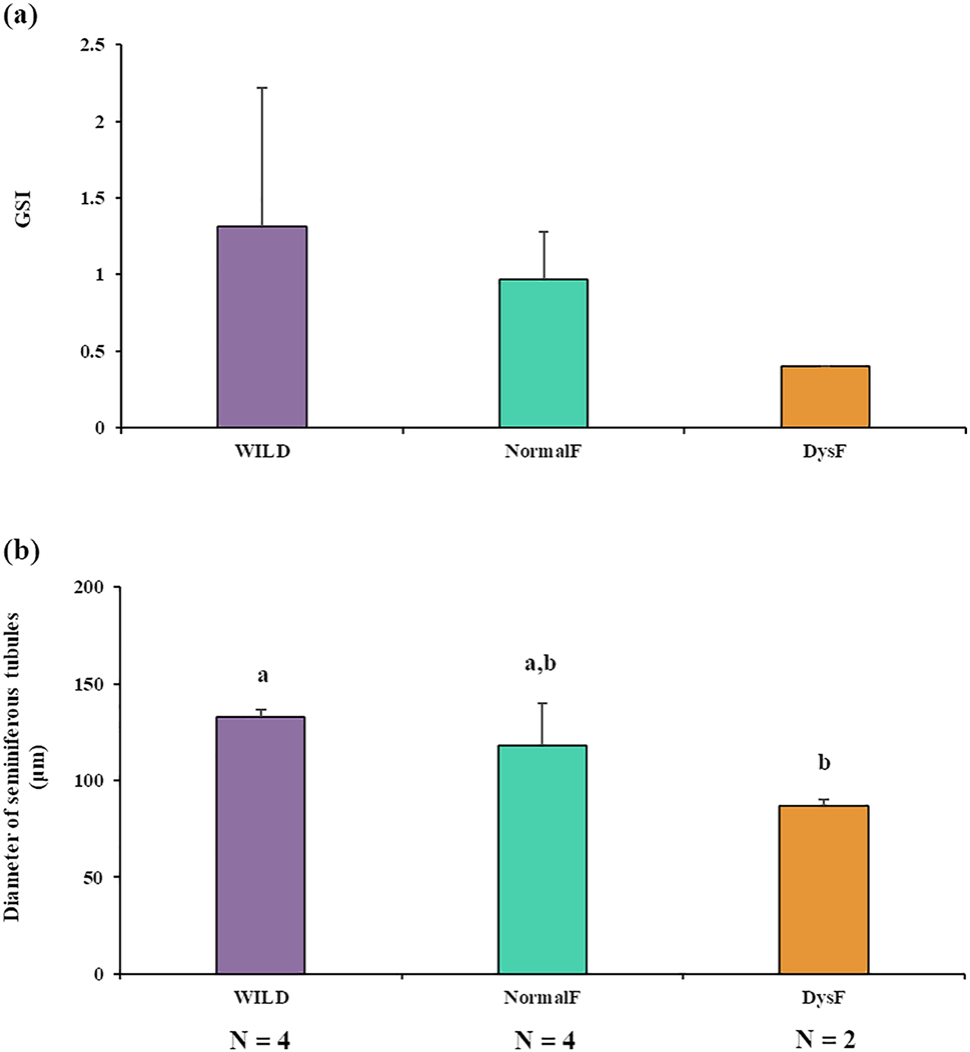
Gonadosomatic index (**a**) and seminiferous tubule diameter (**b**) of wild greater amberjack (WILD), non-dysfunctional farmed fish (NormalF) and dysfunctional farmed fish (DysF). Different letters indicate statistically significant differences (Student’s t-test; P < 0.05).

Wild greater amberjack had testes in active spermatogenesis, showing germ cell in all stages of gametogenesis, seminiferous tubules with large lumen and abundant luminal spermatozoa (Fig. 2a, b). Among farmed fish, two different sub-groups were identified: four individuals had testes in active spermatogenesis similar to wild fish, as evidenced from their histological appearance (Fig. 2c, d), GSI and seminiferous tubule diameter (Fig. 3); and two individuals that showed evident reproductive dysfunction characterized by reduced spermatogenic activity, seminiferous tubules with smaller lumen and limited amount of spermatozoa (Fig. 2e, f), lower GSI and smaller diameter of seminiferous tubules (Fig. 3).

The comparative RNA-seq analysis was performed on the following groups of fish: wild fish (WILD; all of them with normal spermatogenic activity; N = 3); non-dysfunctional farmed fish showing apparently normal spermatogenic activity (NormalF; N = 4); reproductively dysfunctional farmed fish (DysF; N = 2).

### Transcriptome analysis

The testis comparative transcriptome analysis among the three groups of fish in different reproductive conditions (WILD, NormalF and DysF) produced an average of 25 million paired-end reads per sample. After an appropriate cleaning procedure, high quality reads were aligned to the *Seriola dumerili* reference genome. About the 90% of cleaned reads were uniquely mapped to the reference genome.

**Figure 4:**
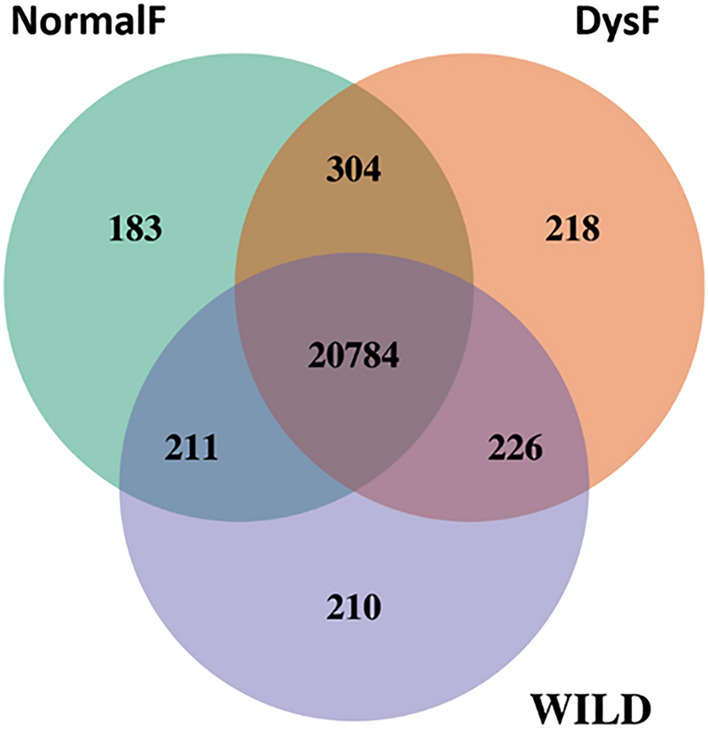
VENN diagram of shared and unique genes related to testes samples of wild (WILD) and hatchery-produced greater amberjack, dysfunctional (DysF) and non-dysfunctional (NormalF).

The comparative transcriptome analysis showed that the majority of the expressed genes (20784) (with a normalized reads count of at least 10) were common to the three groups, while only 210, 183 and 218 genes were specifically expressed in WILD, NormalF and DysF respectively (Fig. 4).

**Figure 5:**
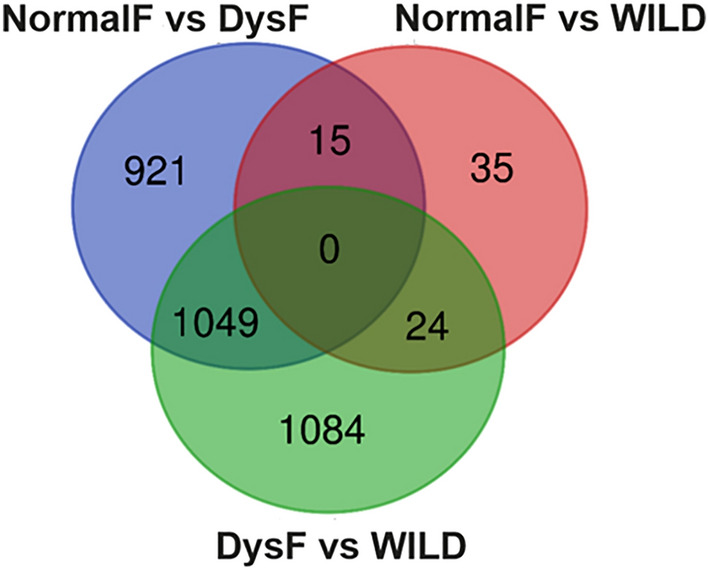
VENN diagram of DEGs shared and unique among DysF vs NormalF, DysF vs WILD and NormalF vs WILD comparisons. DysF, dysfunctional farmed; NormalF, non-dysfunctional Farmed; WILD, wild greater amberjack.

Differential gene expression analysis identified 2157, 1985 and 74 DEGs in DysF vs WILD, NormalF *vs* DysF and NormalF *vs* WILD comparisons, respectively. Among the DEGs, 24 were common to the comparisons DysF *vs* WILD and NormalF *vs* WILD, 15 were common to the comparisons DysF *vs* NormalF and NormalF *vs* WILD, whereas 1049 were common to the comparisons DysF *vs* NormalF and DysF *vs* WILD (Fig. 5; Supplementary Table S1).

Biological categories related to gene ontology enrichment analysis performed on DEGs of each comparison are reported in Table 2.

**Table 2:** Gene ontology enrichment analysis of DEGs from wild and hatchery-produced greater amberjack testes.

	Category	Term	Count	FDR
DysF versus NormalF	UP_KW_BIOLOGICAL_PROCESS	KW-0970~Cilium biogenesis/degradation	12	2.23E−05
	UP_KW_CELLULAR_COMPONENT	KW-0966~Cell projection	39	1.19E−13
	UP_KW_CELLULAR_COMPONENT	KW-0969~Cilium	18	2.13E−10
	UP_KW_CELLULAR_COMPONENT	KW-0206~Cytoskeleton	36	4.76E−07
	UP_KW_CELLULAR_COMPONENT	KW-0963~Cytoplasm	114	2.79E−04
	UP_KW_CELLULAR_COMPONENT	KW-0493~Microtubule	18	4.38E−04
	UP_KW_CELLULAR_COMPONENT	KW-0243~Dynein	5	3.24E−02
	UP_KW_CELLULAR_COMPONENT	KW-0282~Flagellum	5	4.09E−02
	UP_KW_MOLECULAR_FUNCTION	KW-0808~Transferase	85	2.48E−02
	KEGG_PATHWAY	sdu04914:ProgesteronE−mediated oocyte maturation	21	6.69E−03
	KEGG_PATHWAY	sdu04114:Oocyte meiosis	24	6.69E−03
	KEGG_PATHWAY	sdu04110:Cell cycle	23	3.42E−02
DysF versus WILD	UP_KW_BIOLOGICAL_PROCESS	KW-0970~Cilium biogenesis/degradation	10	4.39E−03
	UP_KW_CELLULAR_COMPONENT	KW-0966~Cell projection	30	2.07E−06
	UP_KW_CELLULAR_COMPONENT	KW-0969~Cilium	14	9.60E−06
	UP_KW_CELLULAR_COMPONENT	KW-0206~Cytoskeleton	32	2.48E−04
	UP_KW_CELLULAR_COMPONENT	KW-0493~Microtubule	18	1.52E−03
NormalF versus WILD	KEGG_PATHWAY	sdu04145:Phagosome	6	1.74E−02

Count indicates DEGs belonging to specific category. FDR (False Discovery Rate) are statistically significant (P < 0.05), corrected by the Benjamini–Hochberg procedure describing the level of significance of the enrichment. Term column corresponds to the denomination provided by the DAVID annotation tool.

DysF, dysfunctional farmed group; NormalF, normal farmed group; WILD, wild group.

**Figure 6:**
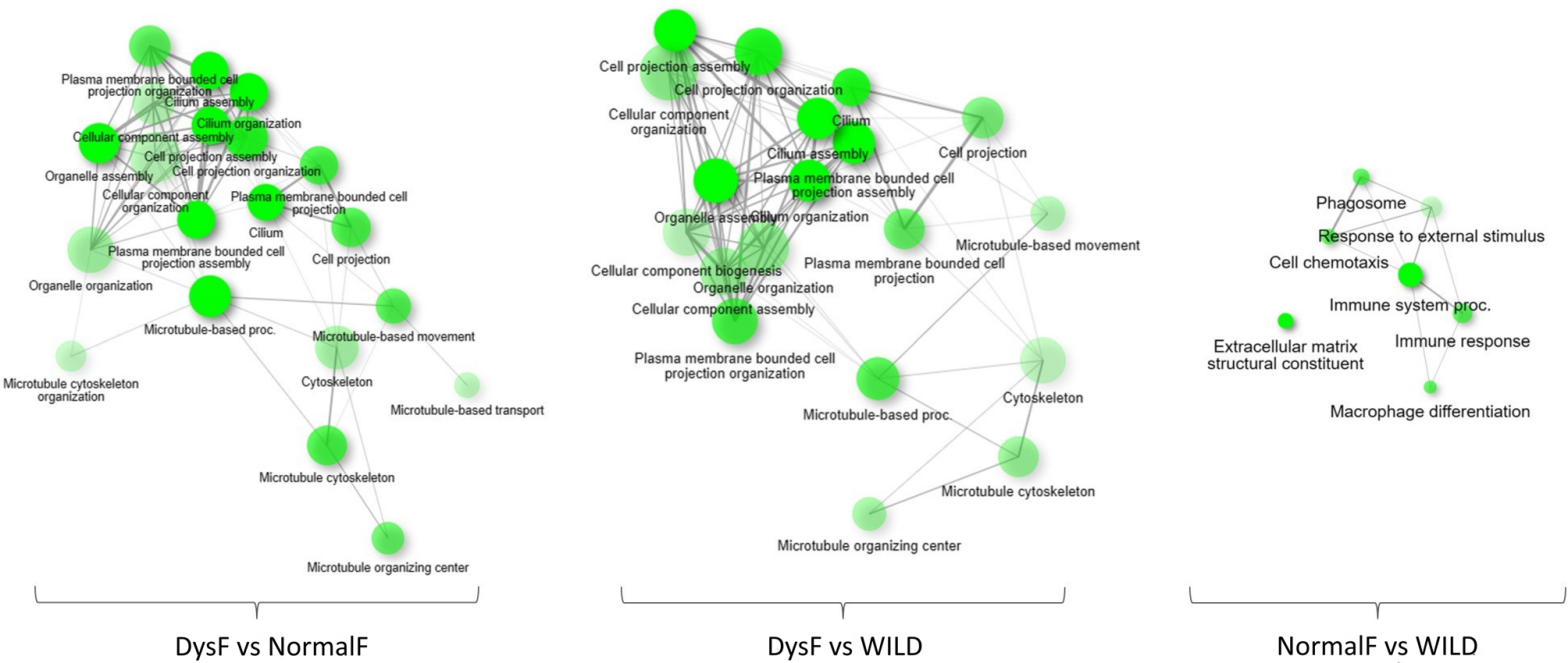
Relationship between enriched pathways in testis samples from the three analysed groups. Pathways (nodes) are connected if they share 20% (default) or more genes. Darker nodes are more significantly enriched gene sets. Bigger nodes represent larger gene sets. Thicker edges represent more overlapped genes.

In general, a statistically significant gene enrichment of biological processes and cellular components related to *cilium* was found in DysF *vs* NormalF as well as in DysF *vs* WILD comparisons. Enriched KEGG pathways, related to *progesterone-mediated oocyte maturation*, *oocyte meiosis* and *cell cycle,* were further identified in DysF *vs* NormalF comparison, while a unique enriched KEGG pathway associated to *phagosome* emerged when NormalF was compared to WILD. The connections between enriched categories are showed in Fig. 6.

In the three comparisons all the enriched categories were interconnected, except for the *extracellular matrix structural constituent* in NormalF *vs* WILD.

**Figure 7:**
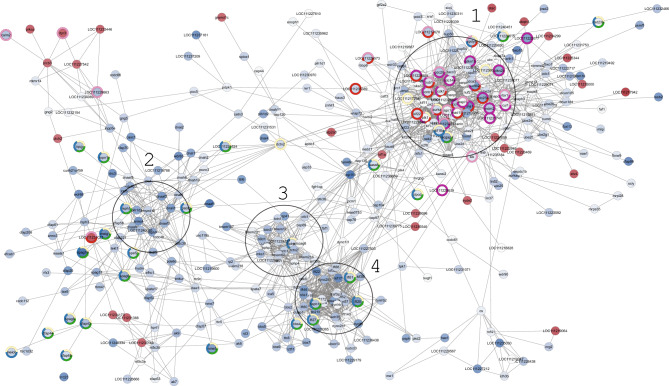
Protein–Protein interaction (PPi) network in DysF *vs* NormalF. The network was built using a confidence protein interaction (score = 0.7). Node background indicates gene upregulation (red, log2FC > 1.5) or downregulation (blue, log2FC < 1.5). Node contour indicates biological categories: *male gamete generation* (blue), *gamete generation* (yellow) *spermatogenesis* (green); KEGG: *progesterone-mediated oocyte maturation* (red), o*ocyte meiosis* (pink), *cell cycle* (purple) pathways. Circles 1–4 are arbitrary representations of the main protein-protein interaction groups.

**Figure 8:**
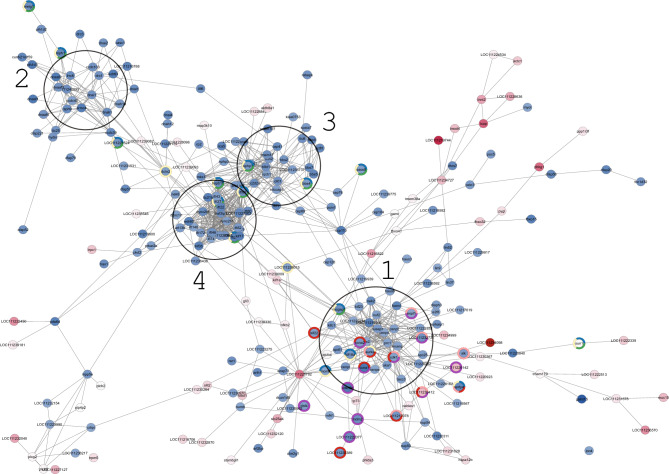
Protein–Protein interaction (PPi) network in DysF *vs* WILD. The network was built using a confidence protein interaction (score = 0.7). Node background indicates gene upregulation (red, log2FC > 1.5) or downregulation (blue, log2FC < 1.5). Node contour indicates biological categories: *male gamete generation* (blue), *gamete generation* (yellow) *spermatogenesis* (green), KEGG: *Progesterone-mediated oocyte maturation* (red), KEGG: *Oocyte meiosis* (pink) KEGG: *Cell cycle* (purple). Circles 1–4 are arbitrary representations of the main protein-protein interaction groups.

**Figure 9:**
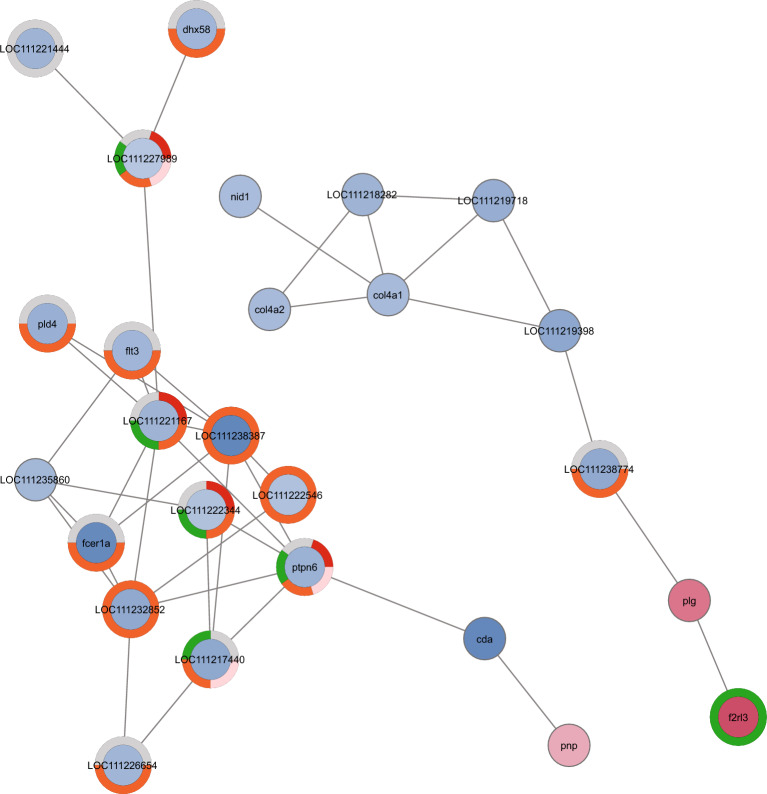
Protein–Protein interaction (PPi) networks in NormalF *vs* WILD. Networks were built using a medium confidence protein interaction (score = 0.4). Nodes are coloured according to the following biological categories: *regulation of tumor necrosis factor production* (red); *cell killing* (pink); *interspecies interaction between organisms* (gray); *sequestering of calcium ion* (green); *immune system process* (orange).

To evaluate the functional relations between DEGs for each comparison, a network based on Protein–Protein interaction (PPi) was generated (Figs. 7, 8 and 9; Supplementary Tables S2a, b and c). Four main protein-interaction groups emerged both in DysF vs NormalF and DysF *vs* WILD comparisons. Most of these proteins were associated to biological processes related to *cilium organization*, *cilium assembly*, *plasma membrane bounded cell projection assembly*, *organelle assembly*, *cellular component organization* and to *cell cycle*. Proteins related to *male gamete generation*, *gamete generation*, *spermatogenesis* (Spdya, Bbs4, Racgap1, Ift81, Ift20, Ift27, Hspb11, Nphp1, Plk1) as well as to the following KEGG pathways were also detected in both comparisons: *progesterone-mediated oocyte maturation* (Igf-1, Cdc), *oocyte meiosis* (AurkA, CycB) and *cell cycle* (CycA) (contour-coloured nodes in Figs. 7 and 8). Moreover, several proteins, involved in steroid synthesis (such as Cholesterol 25-hydroxylase like 3 and Cytochrome P450), as well as several heat shock proteins, were identified in addition to those included in PPi networks (Supplementary Table S2a, b).

Two main protein-interaction groups emerged from the comparison NormalF *vs* WILD. These proteins were associated to the *regulation of tumor necrosis factor production* and *cell killing* (Ptpn6), *interspecies interaction between organisms* (Fcer1a) *immune system* (Flt3) and *regulation of sequestering and release of calcium ion* (F2rl3) (Fig. 9; Supplementary Table S2c).

Gene expression levels related to genes detected in NormalF *vs* DysF and DysF *vs* WILD, as well as those observed in NormalF *vs* WILD were evaluated. Most of the DEGs involved in the biological categories *male gamete generation* and *spermatogenesis,* and KEGG pathways *progesterone-mediated oocyte maturation* (*igf*1, *cdc)*, *oocyte meiosis* (*aurka, cycb*) and *cell cycle* (*cyca*) as well as the heat shock protein gene *hsbp*1 were downregulated in DysF compared with the two non-dysfunctional groups. On the contrary, DEGs belonging to the sterol desaturase family (e.g., LOC111231653: cholesterol 25-hydroxylase like 3) were upregulated in DysF. As shown in Fig. 9, most of the DEGs of the NormalF group were downregulated compared with the WILD group, except the gene *f2rl*3.

Since previous studies showed an increase of germ cell apoptosis in reproductively dysfunctional fish^7,19,27^, DEGs encoding for proteins involved in the *apoptosis* pathway were investigated in all comparisons and they were identified in DysF *vs* NormalF (N = 7) (Table 3; Supplementary Fig. S1), and DysF *vs* WILD (N = 17) (Table 3; Supplementary Fig. S2).

**Table 3:** Dysregulated genes associated to apoptosis.

Protein ID	Gene Entrez ID	KEGG identifier (K) product	DF vs NF		DF vs WILD	
			log2(FC)^a^	padj^b^	log2(FC)	padj
IAP/XIAP	111220232	K08731 baculoviral IAP repeat-containing protein 5-like	− 2.34	9.47E−04	− 2.04	2.36E−03
CytC	111223891	K08738 cytochrome c	− 2.94	9.47E−04	− 2.77	1.15E−03
α-tubulin	111230200	K07374 tubulin alpha-1C chain-like	− 2.26	3.69E−03	− 1.62	4.03E−02
Akt/PKB	111235389	K04456 RAC-gamma serine/threoninE−protein kinase	− 2.07	2.01E−02	− 1.97	3.54E−02
Cathepsin	111231108	K08568 cathepsin Z-like	− 2.18	1.04E−02	–	–
Bcl-XL	111234723	K04570 bcl2l1; bcl-2-like protein 1	− 1.58	2.50E−02	–	–
IP3R	111235419	K04960 itpr3; inositol 1,4,5-trisphosphate receptor type 3	1.68	3.12E−02	–	–
Lamin	111220840	K12641 lamin-A-like	–	–	− 2.75	4.08E−02
Perforin	111216537	K07818 perforin-1-like	–	–	2.12	2.05E−02
α-tubulin	111218500	K07374 tubulin alpha chain-like	–	-	1.88	2.77E−02
Calpain	111220564	K01367 calpain-1 catalytic subunit-like isoform X1	–	-	1.87	3.16E−02
Calpain	111220638	K03853 calpain-2 catalytic subunit-like	–	-	3.81	5.02E−03
Cathepsin	111220923	K01366 pro-cathepsin H-like	–	-	1.92	9.42E−03
Gaad45	111226142	K04402 growth arrest and DNA damage-inducible protein GADD45 beta-like	–	–	2.45	1.82E−03
Cathepsin	111230367	K01379 cathepsin D-like	–	–	1.76	8.16E−03
Cathepsin	111234098	K01371 cathepsin L1-like	–	–	4.81	1.19E−02
Calpain	111237555	K03853 calpain-2 catalytic subunit-like	–	–	2.37	1.82E−03
Calpain	111237566	K01367 calpain-1 catalytic subunit-like	–	–	1.61	1.36E−02
IκBα	111238330	K04734 NF-kappa-B inhibitor alpha-like	–	–	1.61	9.76E−03
Perforin	111239867	K07818 perforin-1-like	–	–	2.11	3.27E−02

^a^log2(FC)|> 1.5 upregulated gene, log2(FC)|< 1.5 downregulated gene; ^b^Bonferroni or Benjamini adjusted P value < 0.05.

In DysF *vs* NormalF, except for gene encoding for inositol 1,4,5-trisphosphate receptor type 3 (*itpr*3) all other DEGs were downregulated. In DysF *vs* WILD, 5 DEGs were downregulated while 12 were upregulated. Downregulated genes coding for the baculoviral IAP repeat-containing protein 5-like (IAP/XIAP), cytochrome c (CytC), tubulin alpha-1C chain-like (α tubulin) and RAC-gamma serine/threonine-protein kinase (Akt/PKB) were identified both in DysF *vs* NormalF and DysF *vs* WILD.

## Discussion

Thanks to the integration of histological and RNA-seq data, this comparative study on wild *vs* hatchery-produced greater amberjack males provided new information on the molecular mechanisms underlying the spermatogenesis impairment observed in fish reared in captivity. Based on testicular development (GSI and histological appearance), hatchery-produced greater amberjack males in the present study were affected by a reproductive dysfunction similar to that displayed by individuals taken from the wild as juveniles and reared in captivity to reproductive maturity^12,19^. Although the number of dysfunctional fish was low due to the limited availability of farmed fish, which belonged to a small broodstock produced in captivity by hormonal induction of spawning, many significantly differentially expressed genes were identified in dysfunctional fish. Moreover, the comparative transcriptome analysis suggested that spermatogenesis was abnormal also in the histologically evaluated “normal” hatchery-produced fish, which might have been at an initial stage of a reproductive dysfunction.

Hereafter, for the sake of clarity, data interpretation and discussion will be referred to the comparison between DysF and WILD group if not further specified. The comparative analysis of RNA-seq data showed that 20784 genes were expressed in all the three groups and about 10% of these genes were differentially expressed between farmed fish showing clear reproductive dysfunction (DysF), and fish that did not show evident gametogenesis alteration (NormalF and WILD).

Among the mapped pathways, DEGs involved in “cell cycle”, “progesterone-mediated oocyte maturation” and “oocyte meiosis” were mostly downregulated in reproductively dysfunctional fish. The pathway “cell cycle” includes genes involved in cell proliferation. The pathways “progesterone-mediated oocyte maturation” and “oocyte meiosis”, despite the names they have been assigned, include genes related to both oogenesis and spermatogenesis processes; e.g., *spyda* encodes for a cell cycle regulator that plays a role in male germ cell meiotic maturation^43^; *igf*1 is associated with testicular activation by recombinant growth hormone in rats^44^ and with testicular germ cell proliferation and apoptosis in fish^45^*; aurka* is required for male germline maintenance and regulates sperm motility in mice^46^.

It is known that the reproductive dysfunctions occurring in fish reared in captivity result from a reduced pituitary release of gonadotropins, particularly Lh that is mainly involved in gamete maturation and ovulation/spermiation via 17,20β-dihydroxy-4-pregnen-3-one (17,20β-P) synthesis^1,2,23,47^. Nevertheless, it is likely that the observed dysregulation of genes involved in *steroid synthesis*, *cell cycle* and *meiosis* may originate from the low levels of plasma Lh in hatchery-produced greater amberjack. Reduced sperm production associated with low levels of plasma Lh has been demonstrated in striped bass *Morone saxatilis* reared in captivity, in comparison with wild fish sampled in their spawning grounds^23,24,48^. Most of the identified DEGs encode for products associated with cellular components “cilium”, “cytoskeleton”, “microtubule”, “flagellum”, “microtubule”, “dynein”. The downregulations of these genes is likely related both to a decreased ability of spermatogonia to enter meiosis and the subsequent spermatid differentiation to spermatozoa. Moreover, several important members of the intraflagellar transport process (*ift*20, *ift*27, *ift*81, etc.) were downregulated. In mice *Mus musculus*, the absence of *ift*81is associated with abnormal flagellum formation and infertility^49^. These findings are coherent with the results of the histological analysis indicating that the dysfunctional fish had a reduced spermatogenic activity and with our previous study showing reduced meiosis in captive-reared greater amberjack^19^.

In agreement with^50^, who reported upregulation of glycolytic enzymes and downregulation of tricarboxylic acid cycle (TCA) and mitochondrial oxidative phosphorylation enzymes in gilthead seabream *Sparus aurata* ejaculated spermatozoa compared with diploid germ cells, we found downregulation of several genes belonging to these metabolic pathways. This finding is coherent with a reduced sperm production in dysfunctional fish. As expected, all of the enriched categories were interconnected, as they were all associated with the process of germ cell division and differentiation, except for the extracellular matrix structural constituent which appeared in the comparison between NormalF *vs* WILD. According to^51^, the testicular maturation of the rainbow trout *Onchorhynchus mykiss* is marked by changes of the expression pattern of genes encoding extracellular matrix proteins and this observation was supposed to be correlated with the reorganization of seminiferous tubules occurring during the testicular cycle^42^.

The PPi analysis confirmed that most of the proteins encoded by DEGs were associated to biological processes related to *spermatogenesis*, *gamete maturation*, *meiosis*, *cell cycle* and *cell assembly*. As expected, among DEGs, genes encoding for enzymes involved in steroid synthesis as well as several heat shock proteins were identified. In particular, the upregulation of gene encoding for cholesterol 25-hydroxylase like 3 is likely associated to stress-induced cortisol synthesis rather than sex steroid synthesis, since sex steroid secretion has been found to be compromised in greater amberjack confined in captivity^12,19^. This interpretation is supported by the upregulation of genes encoding for heat shock proteins, which are known biomarkers of fish exposure to stress^52^, as well as downregulation of genes encoding for CyP450, one of the main enzymes complexes involved in steroidogenesis, and insulin-like growth factors, known mediators of Fsh in the stimulation of spermatogenesis.

Further interesting information originated from the NormalF *vs* WILD PPi comparison that showed dysregulation of genes associated with tumor necrosis factor production, a cytokine produced by leukocytes and involved in inflammation, apoptosis signalling and cell killing. In particular, the *ptpn*6 gene, which encodes for a key regulatory protein involved in different pathways related to inflammation, apoptosis and necroptosis^53^, was differently expressed only in the NormalF *vs* WILD comparison. This gene has a protective role in the regulation of apoptosis^54^ and its downregulation in the NormalF group is coherent with the increased testicular apoptosis observed in captive-reared greater amberjack undergoing an apparently “normal” spermatogenic process^19^.

Some genes encoding regulatory factors involved in the apoptotic process (e.g. aculoviral IAP repeat-containing protein 5-like, cytochrome c, RAC-gamma serine/threonine-protein kinase) were downregulated in DysF *vs* NormalF, whereas other genes encoding for enzymes involved in the apoptotic process (e.g. cathepsin, calpain) were downregulated in DysF *vs* WILD, indicating an overall dysregulation of the apoptotic pathway in the two farmed groups. In the present study, several genes encoding for enzymes involved in the apoptotic process were downregulated in full-blown dysfunctional fish, indicating that when the spermatogenesis is arrested, the role of apoptosis in the removal of aberrant cells and assuring the correct germ cells/Sertoli cells ratio declines. In the DysF vs NormalF comparison, the only upregulated gene involved in the apoptotic process was *itpr*3, a pleiotropic gene which enables Ca2+ transfer from the endoplasmic reticulum to mitochondria, plays a role in metabolism and cell fate regulation and promotes either cell death or cell cycle progression and proliferation^55^. Hence, the upregulation of this gene in dysfunctional fish might be related to an increased germ cells apoptosis.

The downregulation of genes encoding for proteins involved in interspecies interaction between organisms and immune response in apparently non-dysfunctional fish may be interpreted as a further evidence of the inflammatory state induced by captivity-induced stress; furthermore, it may also suggest a reduced synthesis of factors involved in sperm-oocyte interaction.

In conclusion, 30% of the analysed hatchery-produced greater amberjack showed the same reproductive dysfunction previously observed in individuals caught from the wild and reared in captivity. Moreover, the presence of statistically significant differences in gene expression and disrupted pathways suggested that even apparently non-dysfunctional fish might have been experiencing an initial stage of reproductive impairment. The molecular mechanisms generating the observed spermatogenesis alteration involved dysregulation of many interconnected biological processes, such as steroidogenesis, cell cycle, meiosis, cell assembly, and apoptosis. Further studies are in progress on gene expression in pituitary and hypothalamus in order to provide a complete view of the alteration of the activity of the reproductive axis in greater amberjack reared under commercial farming conditions. The identification of the altered biological processes in fish reared in captivity will improve our understanding of the observed reproductive dysfunctions and will hopefully support the set-up of more effective broodstock management protocols in the aquaculture industry.

### Supplementary Information

Supplementary Figures.

Supplementary Tables.

## Data Availability

Reads generated in this study are freely available through the SRA (Short Read Archive) database under the BioProject accession number PRJNA946197. All the other data produced and/or analyzed during the current study are included in this article and in Supplementary Figures and Supplementary Tables.
