# Supplementary material for: Dysregulation of testis mRNA expression levels in hatchery-produced vs wild greater amberjack Seriola dumerili
Source: Sci Rep. 2023 Aug 22;13:13662. doi: 10.1038/s41598-023-40597-5 (PMC10444852; doi:10.1038/s41598-023-40597-5)
Supplement: Supplementary file 1 — Supplementary Figures. [file 41598_2023_40597_MOESM1_ESM.docx]

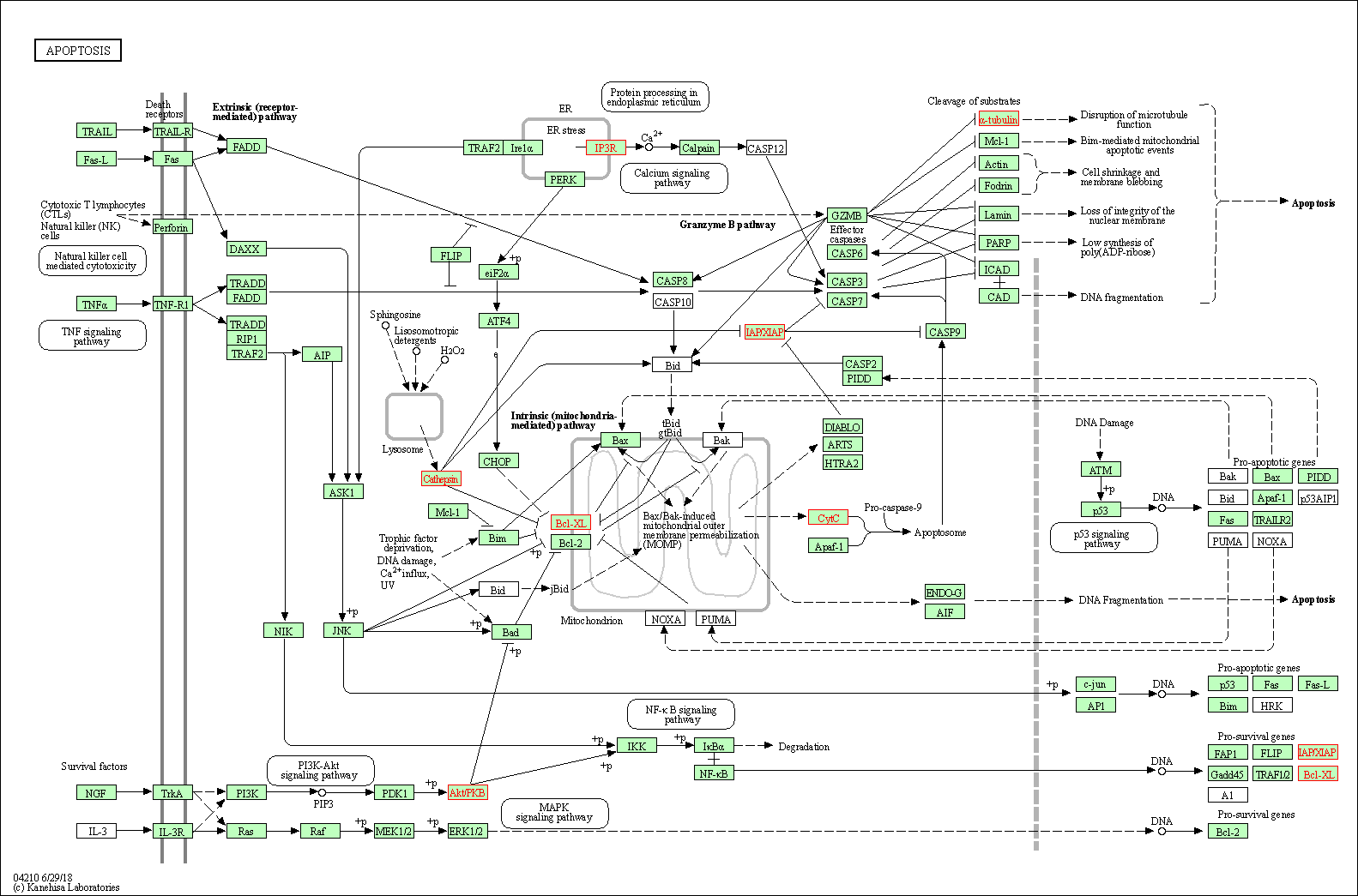


Figure S1. Differentially expressed genes encoding for proteins involved in apoptosis pathway (DysF vs NormalF comparison). Image generated through KEGG Mapper. Generated through KEGG Mapper – Search (<https://www.genome.jp/kegg/mapper/search.html>) [42].


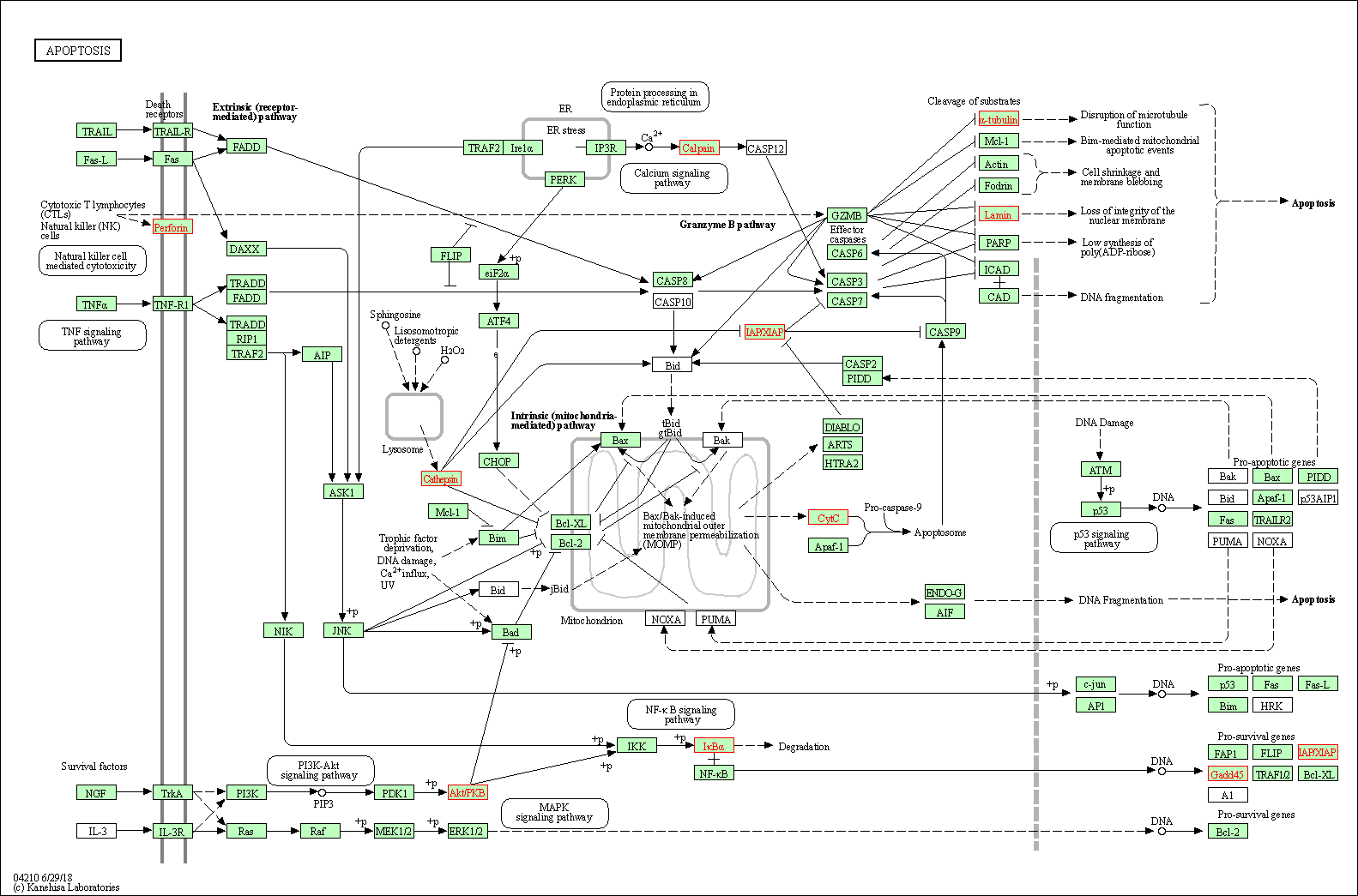


Figure S2. Differentially expressed genes encoding for proteins involved in apoptosis pathway (DysF vs WILD comparisons). Generated through KEGG Mapper – Search (<https://www.genome.jp/kegg/mapper/search.html>) [42].
